# Crystal structure of (*E*)-1-(4-*tert*-butyl­phen­yl)-2-(4-iodo­phen­yl)ethene

**DOI:** 10.1107/S2056989015007185

**Published:** 2015-04-15

**Authors:** Zhiwei Chen, Graeme J. Moxey

**Affiliations:** a School of Chemical and Material Engineering, Jiangnan University, Wuxi, 214122, People’s Republic of China; b Research School of Chemistry, Australian National University, Canberra, ACT 2601, Australia

**Keywords:** crystal structure, stilbene, iodo­arene, C—I⋯π inter­actions

## Abstract

The title compound, C_18_H_19_I, crystallized with two independent mol­ecules (*A* and *B*) in the asymmetric unit. Both mol­ecules have an *E* conformation about the bridging C=C bond. They differ in the orientation of the two benzene rings; the dihedral angle being 12.3 (5)° in mol­ecule *A*, but only 1.0 (6)° in mol­ecule *B*. In the crystal, the individual mol­ecules are linked by C—I⋯π inter­actions forming zigzag *A* and zigzag *B* chains propagating along [001]. The structure was refined as an inversion twin [Flack parameter = 0.48 (2)].

## Related literature   

For the syntheses of aryl­alkynes by Sonogashira cross-coupling of iodo­arenes, see: Takahashi *et al.* (1980 ▸). For desilylation of the resultant tri­alkyl­silylethynylarenes and the use of ethynylarenes in the construction of metal alkynyl complexes with enhanced non-linear optical properties, see: McDonagh *et al.* (1996*a* ▸,*b* ▸, 2003 ▸); Garcia *et al.* (2002 ▸). For related structures, see: Marras *et al.* (2006 ▸); Mariaca *et al.* (2009 ▸).
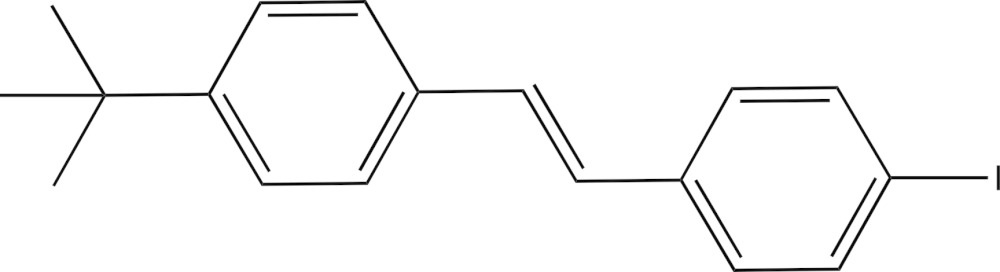


## Experimental   

### Crystal data  

C_18_H_19_I
*M* *_r_* = 362.23
Orthorhombic, 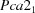

*a* = 32.5385 (9) Å
*b* = 6.10513 (15) Å
*c* = 15.8615 (3) Å
*V* = 3150.91 (14) Å^3^
*Z* = 8
Cu *K*α radiation
μ = 15.83 mm^−1^
*T* = 150 K
0.16 × 0.05 × 0.02 mm

### Data collection  

Agilent SuperNova (Dual, Cu at zero, EosS2) diffractometer
Absorption correction: analytical (*CrysAlis PRO*; Agilent, 2014 ▸) *T*_min_ = 0.854, *T*_max_ = 0.966
10293 measured reflections
3770 independent reflections
3559 reflections with *I* > 2σ(*I*)
*R*_int_ = 0.033

### Refinement  

*R*[*F* ^2^ > 2σ(*F* ^2^)] = 0.050
*wR*(*F* ^2^) = 0.133
*S* = 1.04
3770 reflections
350 parameters
67 restraints
H-atom parameters constrained
Δρ_max_ = 2.09 e Å^−3^
Δρ_min_ = −1.31 e Å^−3^
Absolute structure: Flack (1983 ▸), 570 Friedel pairs
Absolute structure parameter: 0.48 (2)

### 

Data collection: *CrysAlis PRO* (Agilent, 2014 ▸); cell refinement: *CrysAlis PRO*; data reduction: *CrysAlis PRO*; program(s) used to solve structure: *SHELXS97* (Sheldrick, 2008 ▸); program(s) used to refine structure: *SHELXL2013* (Sheldrick, 2015 ▸); molecular graphics: *OLEX2* (Dolomanov *et al.*, 2009 ▸); software used to prepare material for publication: *OLEX2*.

## Supplementary Material

Crystal structure: contains datablock(s) Global, I. DOI: 10.1107/S2056989015007185/su5108sup1.cif

Structure factors: contains datablock(s) I. DOI: 10.1107/S2056989015007185/su5108Isup2.hkl

**Fig. 1.**
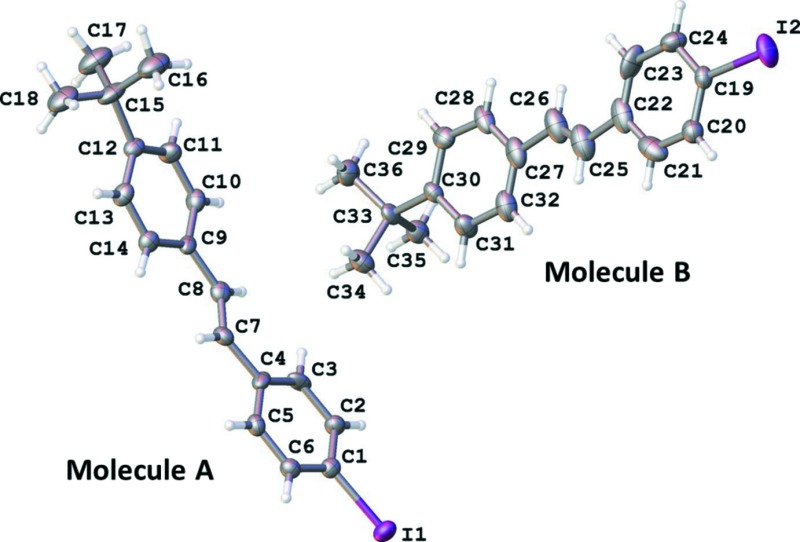
Molecular structure of the two independent molecules (A and B) of the title compound, with atom labelling. Displacement ellipsoids are drawn at the 40% probability level.

**Fig. 2.**
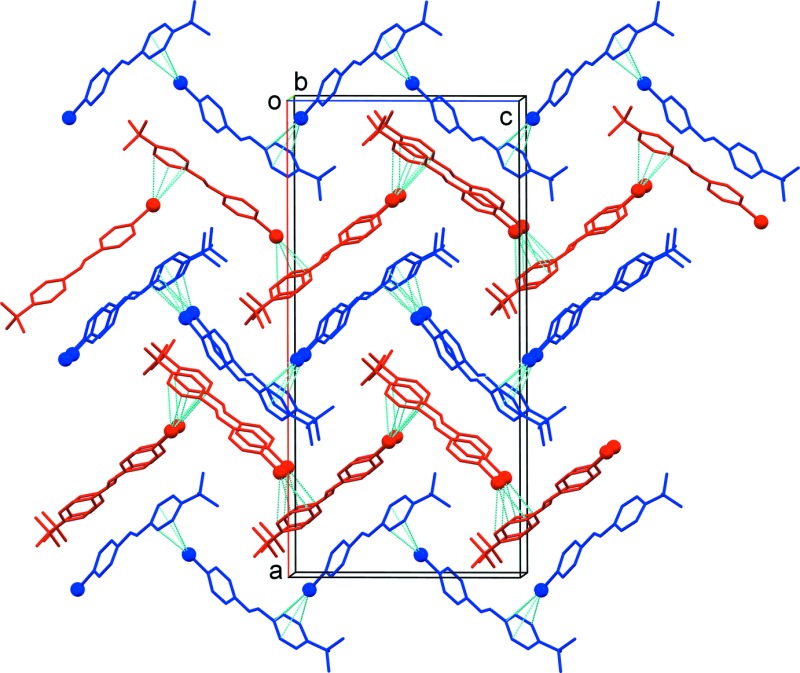
A view along the b axis of the crystal packing of the title compound. The C—I···π interactions are represented as dashed lines (see Table 1 for details; molecule A blue, molecule B red).

A B . DOI: 10.1107/S2056989015007185/su5108fig1.tif
Mol­ecular structure of the two independent mol­ecules (*A* and *B*) of the title compound, with atom labelling. Displacement ellipsoids are drawn at the 40% probability level.

b A B . DOI: 10.1107/S2056989015007185/su5108fig2.tif
A view along the *b* axis of the crystal packing of the title compound. The C—I⋯π inter­actions are represented as dashed lines (see Table 1 for details; mol­ecule *A* blue, mol­ecule *B* red).

**Fig. 3.**
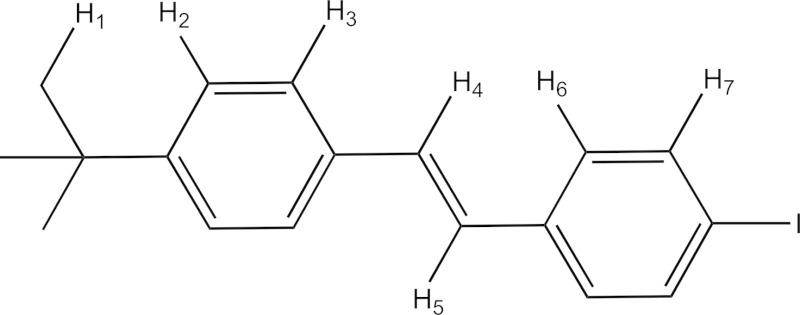
Atom numbering scheme of the title compound for 1H NMR assignments.

1 . DOI: 10.1107/S2056989015007185/su5108fig3.tif
Atom numbering scheme of the title compound for ^1^H NMR assignments.

CCDC reference: 1053466

Additional supporting information:  crystallographic information; 3D view; checkCIF report

## Figures and Tables

**Table 1:** Hydrogen-bond geometry (, ) *Cg*2 and *Cg*4 are the centroids of the C9C14 and C27C32 rings, respectively.

*D*H*A*	*D*H	H*A*	*D* *A*	*D*H*A*
C1I1*Cg*2^i^	2.09(1)	3.63(1)	5.676(10)	166(1)
C19I2*Cg*4^ii^	2.10(1)	3.57(1)	5.526(11)	154(1)

Symmetry codes: (i) 
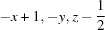
; (ii) 
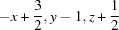
.

## supplementary crystallographic information

### S1. Synthesis and crystallization

(*E*)-1-(4-*tert*-butyl­phenyl)-2-(4-bromo­phenyl)­ethene (1.00 g, 3.17 mmol) was dissolved in distilled THF (40 mL) and cooled to 195 K (liquid nitro­gen bath) under N_2_ for 30 min. BuLi (2.97 mL, 1.6 M, 4.76 mmol) was added and the mixture was stirred for 2 h. A solution of I_2_ (1.20 g, 4.76 mmol) in THF (20 mL) was then added and the reaction was allowed to warm to room temperature. A saturated solution of sodium thio­sulfate (10 mL) and water (20 mL) were then added and the mixture stirred until clear. The mixture was then extracted with CH_2_Cl_2_, stirred over anhydrous MgSO_4_, filtered and taken to dryness to yield the title compound as a yellow solid. The solid was extracted with a small amount of CH_2_Cl_2_ and the extract was passed through a pad of silica with petrol as eluent. The eluate was reduced in volume, affording the title compound as a white solid (yield: 1.0 g, 87%). The numbering scheme of the title compound for the NMR assignments is given in Fig. 3. ^1^H-NMR (400 MHz, CDCl_3_): δ 7.66 (d, *J*_HH_ = 8 Hz, 2H, H_7_), 7.44 (d, *J*_HH_ = 8 Hz, 2H, H_3_), 7.38 (d, *J*_HH_ = 8 Hz, 2H, H_2_), 7.23 (d, *J*_HH_ = 8 Hz, 2H, H_6_), 7.09 (d, *J*_HH_ = 16 Hz, 1H, H_4_), 6.97 (d, *J*_HH_ = 16 Hz, 1H, H_5_), 1.33 (s, 9H, H_1_). Crystals suitable for X-ray diffraction analysis were obtained by slow evaporation of a solution in hexane.

### S2. Refinement

Crystal data, data collection and structure refinement details are summarized below. The H atoms were included in calculated positions and treated as riding: C—H = 0.93 - 0.96 Å with U_iso_(H) = 1.5U_eq_(C) for methyl H atoms and 1.2U_eq_(C) for other H atoms. The structure was refined as an inversion twin: Flack parameter = 0.48 (2). Rigid bond restraints (RIGU) were applied to atoms C15, C16, C17, C18, C22, C25, C26, C27, C28, C32, C33, C34, C35, C36.

### Crystal data

**Table:**

C_18_H_19_I	*D*_x_ = 1.527 Mg m^−^^3^
*M**_r_* = 362.23	Cu *K*α radiation, λ = 1.54184 Å
Orthorhombic, *P**c**a*2_1_	Cell parameters from 4062 reflections
*a* = 32.5385 (9) Å	θ = 2.7–71.7°
*b* = 6.10513 (15) Å	µ = 15.83 mm^−^^1^
*c* = 15.8615 (3) Å	*T* = 150 K
*V* = 3150.91 (14) Å^3^	Needle, colourless
*Z* = 8	0.16 × 0.05 × 0.02 mm
*F*(000) = 1440	

### Data collection

**Table:**

Agilent SuperNova (Dual, Cu at zero, EosS2) diffractometer	3770 independent reflections
Radiation source: sealed X-ray tube, SuperNova (Cu) X-ray Source	3559 reflections with *I* > 2σ(*I*)
Mirror monochromator	*R*_int_ = 0.033
Detector resolution: 8.1297 pixels mm^-1^	θ_max_ = 72.3°, θ_min_ = 3.9°
ω scans	*h* = −36→40
Absorption correction: analytical (*CrysAlis PRO*; Agilent, 2014)	*k* = −7→7
*T*_min_ = 0.854, *T*_max_ = 0.966	*l* = −7→19
10293 measured reflections	

### Refinement

**Table:**

Refinement on *F*^2^	Secondary atom site location: difference Fourier map
Least-squares matrix: full	Hydrogen site location: inferred from neighbouring sites
*R*[*F*^2^ > 2σ(*F*^2^)] = 0.050	H-atom parameters constrained
*wR*(*F*^2^) = 0.133	*w* = 1/[σ^2^(*F*_o_^2^) + (0.0776*P*)^2^ + 7.7934*P*] where *P* = (*F*_o_^2^ + 2*F*_c_^2^)/3
*S* = 1.04	(Δ/σ)_max_ = 0.001
3770 reflections	Δρ_max_ = 2.09 e Å^−^^3^
350 parameters	Δρ_min_ = −1.31 e Å^−^^3^
67 restraints	Absolute structure: Flack (1983), 570 Friedel pairs
Primary atom site location: structure-invariant direct methods	Absolute structure parameter: 0.48 (2)

### Special details

**Table:**

Geometry. All e.s.d.'s (except the e.s.d. in the dihedral angle between two l.s. planes) are estimated using the full covariance matrix. The cell e.s.d.'s are taken into account individually in the estimation of e.s.d.'s in distances, angles and torsion angles; correlations between e.s.d.'s in cell parameters are only used when they are defined by crystal symmetry. An approximate (isotropic) treatment of cell e.s.d.'s is used for estimating e.s.d.'s involving l.s. planes.
Refinement. Refined as a 2-component inversion twin.

### Fractional atomic coordinates and isotropic or equivalent isotropic displacement parameters (Å^2^)

**Table:**

	*x*	*y*	*z*	*U*_iso_*/*U*_eq_	
I1	0.54022 (2)	−0.31330 (10)	0.04996 (5)	0.04638 (19)	
C1	0.4990 (3)	−0.1418 (15)	0.1266 (6)	0.0337 (19)	
C2	0.5086 (3)	0.0691 (15)	0.1547 (7)	0.0366 (19)	
H2	0.5327	0.1383	0.1375	0.044*	
C3	0.4807 (4)	0.1750 (14)	0.2101 (7)	0.041 (2)	
H3	0.4870	0.3146	0.2297	0.049*	
C4	0.4457 (3)	0.0801 (18)	0.2351 (6)	0.038 (2)	
C5	0.4356 (3)	−0.1290 (17)	0.2036 (6)	0.0361 (19)	
H5	0.4110	−0.1953	0.2195	0.043*	
C6	0.4621 (3)	−0.2362 (16)	0.1489 (7)	0.036 (2)	
H6	0.4549	−0.3723	0.1272	0.043*	
C7	0.4158 (3)	0.1806 (16)	0.2943 (7)	0.036 (2)	
H7	0.3932	0.0972	0.3102	0.043*	
C8	0.4191 (3)	0.3824 (17)	0.3267 (6)	0.038 (2)	
H8	0.4402	0.4695	0.3060	0.046*	
C9	0.3926 (3)	0.4803 (15)	0.3914 (5)	0.0314 (18)	
C10	0.4042 (3)	0.6749 (16)	0.4264 (7)	0.040 (2)	
H10	0.4268	0.7489	0.4045	0.048*	
C11	0.3829 (3)	0.7647 (17)	0.4941 (8)	0.043 (2)	
H11	0.3918	0.8970	0.5168	0.051*	
C12	0.3485 (3)	0.6614 (15)	0.5290 (6)	0.035 (2)	
C13	0.3352 (3)	0.4714 (16)	0.4881 (7)	0.040 (2)	
H13	0.3114	0.4025	0.5067	0.048*	
C14	0.3563 (3)	0.3835 (15)	0.4213 (6)	0.037 (2)	
H14	0.3464	0.2575	0.3953	0.044*	
C15	0.3286 (5)	0.743 (2)	0.6106 (8)	0.056 (3)	
C16	0.3539 (6)	0.657 (3)	0.6841 (10)	0.077 (3)	
H16A	0.3519	0.5001	0.6862	0.116*	
H16B	0.3437	0.7178	0.7359	0.116*	
H16C	0.3821	0.6985	0.6768	0.116*	
C17	0.3247 (6)	0.985 (3)	0.6122 (10)	0.077 (4)	
H17A	0.3148	1.0351	0.5586	0.115*	
H17B	0.3510	1.0488	0.6236	0.115*	
H17C	0.3056	1.0266	0.6556	0.115*	
C18	0.2857 (5)	0.652 (3)	0.6225 (10)	0.071 (3)	
H18A	0.2873	0.5082	0.6469	0.107*	
H18B	0.2721	0.6435	0.5689	0.107*	
H18C	0.2704	0.7463	0.6594	0.107*	
I2	0.78793 (2)	−0.28068 (15)	0.94298 (6)	0.0660 (3)	
C19	0.7487 (3)	−0.1295 (17)	0.8555 (7)	0.037 (2)	
C20	0.7411 (3)	−0.230 (2)	0.7792 (8)	0.046 (3)	
H20	0.7518	−0.3680	0.7677	0.055*	
C21	0.7175 (4)	−0.124 (3)	0.7204 (8)	0.064 (4)	
H21	0.7135	−0.1904	0.6684	0.077*	
C22	0.6996 (4)	0.073 (3)	0.7341 (10)	0.063 (4)	
C23	0.7063 (4)	0.169 (2)	0.8102 (12)	0.066 (4)	
H23	0.6939	0.3027	0.8217	0.079*	
C24	0.7315 (4)	0.072 (2)	0.8725 (8)	0.053 (3)	
H24	0.7363	0.1425	0.9236	0.063*	
C25	0.6767 (4)	0.164 (3)	0.6608 (12)	0.072 (3)	
H25	0.6724	0.0661	0.6169	0.086*	
C26	0.6631 (4)	0.339 (3)	0.6495 (11)	0.067 (3)	
H26	0.6681	0.4387	0.6927	0.080*	
C27	0.6392 (3)	0.429 (2)	0.5768 (8)	0.056 (2)	
C28	0.6210 (3)	0.629 (2)	0.5896 (7)	0.049 (2)	
H28	0.6257	0.7007	0.6404	0.059*	
C29	0.5963 (3)	0.7274 (18)	0.5311 (7)	0.043 (2)	
H29	0.5840	0.8609	0.5437	0.051*	
C30	0.5893 (3)	0.6305 (15)	0.4530 (7)	0.0351 (18)	
C31	0.6083 (3)	0.4297 (16)	0.4386 (8)	0.048 (2)	
H31	0.6045	0.3588	0.3873	0.057*	
C32	0.6327 (4)	0.334 (2)	0.5003 (10)	0.057 (3)	
H32	0.6452	0.2004	0.4888	0.069*	
C33	0.5640 (3)	0.7429 (16)	0.3844 (7)	0.0379 (18)	
C34	0.5377 (4)	0.578 (2)	0.3364 (8)	0.055 (3)	
H34A	0.5198	0.5036	0.3752	0.082*	
H34B	0.5551	0.4729	0.3091	0.082*	
H34C	0.5215	0.6532	0.2949	0.082*	
C35	0.5934 (4)	0.8569 (19)	0.3232 (8)	0.047 (2)	
H35A	0.6122	0.7514	0.3003	0.070*	
H35B	0.6085	0.9683	0.3526	0.070*	
H35C	0.5780	0.9226	0.2783	0.070*	
C36	0.5344 (4)	0.911 (2)	0.4202 (9)	0.055 (3)	
H36A	0.5171	0.8429	0.4616	0.083*	
H36B	0.5177	0.9693	0.3756	0.083*	
H36C	0.5497	1.0278	0.4460	0.083*	

### Atomic displacement parameters (Å^2^)

**Table:**

	*U*^11^	*U*^22^	*U*^33^	*U*^12^	*U*^13^	*U*^23^
I1	0.0447 (3)	0.0457 (3)	0.0487 (3)	0.0059 (2)	0.0084 (3)	−0.0103 (3)
C1	0.037 (5)	0.033 (4)	0.032 (4)	0.003 (4)	0.000 (4)	0.005 (4)
C2	0.038 (4)	0.032 (4)	0.040 (5)	0.000 (4)	−0.002 (4)	−0.002 (4)
C3	0.058 (6)	0.019 (4)	0.045 (6)	0.001 (4)	−0.020 (5)	−0.007 (4)
C4	0.035 (5)	0.058 (6)	0.021 (4)	0.014 (4)	0.003 (4)	−0.001 (4)
C5	0.029 (4)	0.042 (5)	0.037 (5)	−0.003 (4)	−0.003 (4)	0.004 (4)
C6	0.038 (5)	0.033 (4)	0.036 (5)	−0.001 (4)	−0.003 (4)	0.000 (4)
C7	0.036 (5)	0.043 (5)	0.030 (5)	−0.007 (4)	−0.004 (4)	−0.002 (4)
C8	0.039 (5)	0.043 (5)	0.032 (4)	0.003 (4)	−0.003 (4)	0.010 (4)
C9	0.029 (4)	0.044 (5)	0.020 (4)	0.007 (4)	0.001 (3)	0.005 (3)
C10	0.032 (4)	0.048 (5)	0.039 (5)	0.000 (4)	0.005 (4)	0.010 (4)
C11	0.045 (5)	0.036 (4)	0.048 (6)	−0.007 (4)	0.004 (5)	−0.007 (4)
C12	0.042 (5)	0.038 (4)	0.026 (5)	0.008 (4)	0.004 (4)	0.005 (4)
C13	0.037 (4)	0.037 (4)	0.044 (5)	−0.002 (4)	0.011 (4)	0.005 (4)
C14	0.041 (5)	0.032 (4)	0.038 (5)	0.005 (4)	0.001 (4)	0.006 (4)
C15	0.072 (6)	0.056 (5)	0.040 (5)	0.019 (4)	0.023 (4)	−0.003 (4)
C16	0.096 (7)	0.086 (8)	0.049 (5)	0.027 (6)	0.016 (5)	−0.004 (5)
C17	0.097 (8)	0.065 (5)	0.069 (8)	0.019 (5)	0.034 (7)	−0.003 (4)
C18	0.080 (6)	0.082 (7)	0.052 (7)	0.014 (5)	0.027 (5)	−0.001 (6)
I2	0.0478 (4)	0.0800 (5)	0.0701 (6)	−0.0002 (4)	−0.0213 (4)	0.0277 (5)
C19	0.029 (4)	0.041 (5)	0.040 (5)	0.001 (4)	−0.001 (4)	0.007 (4)
C20	0.040 (6)	0.052 (6)	0.046 (6)	0.000 (5)	−0.002 (5)	0.002 (5)
C21	0.067 (8)	0.089 (10)	0.036 (6)	−0.029 (8)	−0.001 (5)	0.006 (7)
C22	0.037 (5)	0.082 (8)	0.069 (7)	−0.017 (5)	−0.003 (5)	0.039 (6)
C23	0.057 (7)	0.040 (6)	0.101 (13)	0.023 (5)	0.018 (8)	0.025 (7)
C24	0.063 (7)	0.049 (6)	0.047 (6)	0.006 (5)	0.005 (6)	−0.011 (5)
C25	0.051 (6)	0.086 (6)	0.079 (7)	−0.014 (4)	−0.011 (5)	0.037 (5)
C26	0.047 (5)	0.082 (5)	0.072 (6)	−0.016 (4)	−0.013 (5)	0.034 (4)
C27	0.034 (4)	0.076 (5)	0.058 (4)	−0.014 (4)	−0.003 (3)	0.026 (4)
C28	0.035 (4)	0.076 (5)	0.036 (4)	−0.012 (4)	−0.003 (4)	0.024 (4)
C29	0.039 (5)	0.051 (5)	0.039 (6)	−0.008 (4)	0.008 (4)	0.002 (4)
C30	0.034 (4)	0.037 (4)	0.034 (5)	−0.006 (4)	−0.007 (4)	0.004 (4)
C31	0.051 (5)	0.036 (4)	0.056 (6)	0.000 (4)	−0.013 (5)	−0.002 (5)
C32	0.041 (5)	0.065 (6)	0.066 (5)	−0.005 (4)	−0.008 (4)	0.023 (4)
C33	0.040 (4)	0.036 (3)	0.037 (4)	0.006 (3)	−0.016 (3)	0.003 (3)
C34	0.059 (5)	0.057 (5)	0.048 (5)	−0.007 (4)	−0.015 (4)	−0.002 (4)
C35	0.051 (4)	0.042 (4)	0.046 (5)	0.004 (4)	−0.009 (4)	0.003 (4)
C36	0.053 (5)	0.054 (5)	0.059 (6)	0.014 (4)	−0.008 (4)	0.003 (4)

### Geometric parameters (Å, º)

**Table:**

I1—C1	2.091 (10)	I2—C19	2.099 (10)
C1—C2	1.398 (13)	C19—C20	1.379 (17)
C1—C6	1.379 (14)	C19—C24	1.379 (15)
C2—H2	0.9300	C20—H20	0.9300
C2—C3	1.420 (16)	C20—C21	1.372 (19)
C3—H3	0.9300	C21—H21	0.9300
C3—C4	1.339 (16)	C21—C22	1.35 (2)
C4—C5	1.410 (15)	C22—C23	1.36 (2)
C4—C7	1.485 (14)	C22—C25	1.490 (19)
C5—H5	0.9300	C23—H23	0.9300
C5—C6	1.386 (15)	C23—C24	1.41 (2)
C6—H6	0.9300	C24—H24	0.9300
C7—H7	0.9300	C25—H25	0.9300
C7—C8	1.339 (15)	C25—C26	1.17 (2)
C8—H8	0.9300	C26—H26	0.9300
C8—C9	1.467 (13)	C26—C27	1.494 (18)
C9—C10	1.364 (14)	C27—C28	1.37 (2)
C9—C14	1.402 (13)	C27—C32	1.36 (2)
C10—H10	0.9300	C28—H28	0.9300
C10—C11	1.391 (15)	C28—C29	1.367 (16)
C11—H11	0.9300	C29—H29	0.9300
C11—C12	1.399 (15)	C29—C30	1.391 (15)
C12—C13	1.397 (14)	C30—C31	1.391 (14)
C12—C15	1.532 (14)	C30—C33	1.527 (13)
C13—H13	0.9300	C31—H31	0.9300
C13—C14	1.372 (15)	C31—C32	1.390 (17)
C14—H14	0.9300	C32—H32	0.9300
C15—C16	1.52 (2)	C33—C34	1.526 (15)
C15—C17	1.48 (2)	C33—C35	1.530 (16)
C15—C18	1.51 (2)	C33—C36	1.518 (16)
C16—H16A	0.9600	C34—H34A	0.9600
C16—H16B	0.9600	C34—H34B	0.9600
C16—H16C	0.9600	C34—H34C	0.9600
C17—H17A	0.9600	C35—H35A	0.9600
C17—H17B	0.9600	C35—H35B	0.9600
C17—H17C	0.9600	C35—H35C	0.9600
C18—H18A	0.9600	C36—H36A	0.9600
C18—H18B	0.9600	C36—H36B	0.9600
C18—H18C	0.9600	C36—H36C	0.9600
			
C2—C1—I1	120.2 (7)	C20—C19—I2	119.6 (8)
C6—C1—I1	119.9 (7)	C20—C19—C24	119.7 (10)
C6—C1—C2	119.9 (9)	C24—C19—I2	120.7 (9)
C1—C2—H2	120.9	C19—C20—H20	120.4
C1—C2—C3	118.2 (9)	C21—C20—C19	119.1 (12)
C3—C2—H2	120.9	C21—C20—H20	120.4
C2—C3—H3	119.0	C20—C21—H21	118.2
C4—C3—C2	122.0 (9)	C22—C21—C20	123.6 (14)
C4—C3—H3	119.0	C22—C21—H21	118.2
C3—C4—C5	119.1 (9)	C21—C22—C23	117.1 (12)
C3—C4—C7	124.4 (10)	C21—C22—C25	115.0 (16)
C5—C4—C7	116.5 (9)	C23—C22—C25	127.8 (15)
C4—C5—H5	119.9	C22—C23—H23	118.9
C6—C5—C4	120.2 (9)	C22—C23—C24	122.3 (12)
C6—C5—H5	119.9	C24—C23—H23	118.9
C1—C6—C5	120.3 (9)	C19—C24—C23	118.2 (12)
C1—C6—H6	119.8	C19—C24—H24	120.9
C5—C6—H6	119.8	C23—C24—H24	120.9
C4—C7—H7	117.6	C22—C25—H25	114.7
C8—C7—C4	124.9 (10)	C26—C25—C22	131 (2)
C8—C7—H7	117.6	C26—C25—H25	114.7
C7—C8—H8	116.7	C25—C26—H26	114.8
C7—C8—C9	126.6 (10)	C25—C26—C27	130.5 (18)
C9—C8—H8	116.7	C27—C26—H26	114.8
C10—C9—C8	118.4 (9)	C28—C27—C26	115.9 (14)
C10—C9—C14	117.6 (9)	C32—C27—C26	127.8 (14)
C14—C9—C8	124.0 (9)	C32—C27—C28	116.3 (11)
C9—C10—H10	119.4	C27—C28—H28	118.5
C9—C10—C11	121.2 (9)	C29—C28—C27	123.0 (12)
C11—C10—H10	119.4	C29—C28—H28	118.5
C10—C11—H11	119.1	C28—C29—H29	119.6
C10—C11—C12	121.8 (9)	C28—C29—C30	120.9 (11)
C12—C11—H11	119.1	C30—C29—H29	119.6
C11—C12—C15	121.8 (10)	C29—C30—C33	122.1 (9)
C13—C12—C11	116.0 (9)	C31—C30—C29	116.6 (10)
C13—C12—C15	122.1 (10)	C31—C30—C33	121.2 (10)
C12—C13—H13	119.0	C30—C31—H31	119.7
C14—C13—C12	121.9 (9)	C32—C31—C30	120.5 (12)
C14—C13—H13	119.0	C32—C31—H31	119.7
C9—C14—H14	119.4	C27—C32—C31	122.6 (13)
C13—C14—C9	121.1 (9)	C27—C32—H32	118.7
C13—C14—H14	119.4	C31—C32—H32	118.7
C16—C15—C12	107.8 (10)	C30—C33—C35	108.6 (8)
C17—C15—C12	112.0 (11)	C34—C33—C30	111.2 (9)
C17—C15—C16	112.2 (15)	C34—C33—C35	109.6 (10)
C17—C15—C18	106.5 (13)	C36—C33—C30	112.3 (9)
C18—C15—C12	112.1 (12)	C36—C33—C34	106.1 (10)
C18—C15—C16	106.1 (13)	C36—C33—C35	109.0 (9)
C15—C16—H16A	109.5	C33—C34—H34A	109.5
C15—C16—H16B	109.5	C33—C34—H34B	109.5
C15—C16—H16C	109.5	C33—C34—H34C	109.5
H16A—C16—H16B	109.5	H34A—C34—H34B	109.5
H16A—C16—H16C	109.5	H34A—C34—H34C	109.5
H16B—C16—H16C	109.5	H34B—C34—H34C	109.5
C15—C17—H17A	109.5	C33—C35—H35A	109.5
C15—C17—H17B	109.5	C33—C35—H35B	109.5
C15—C17—H17C	109.5	C33—C35—H35C	109.5
H17A—C17—H17B	109.5	H35A—C35—H35B	109.5
H17A—C17—H17C	109.5	H35A—C35—H35C	109.5
H17B—C17—H17C	109.5	H35B—C35—H35C	109.5
C15—C18—H18A	109.5	C33—C36—H36A	109.5
C15—C18—H18B	109.5	C33—C36—H36B	109.5
C15—C18—H18C	109.5	C33—C36—H36C	109.5
H18A—C18—H18B	109.5	H36A—C36—H36B	109.5
H18A—C18—H18C	109.5	H36A—C36—H36C	109.5
H18B—C18—H18C	109.5	H36B—C36—H36C	109.5
			
I1—C1—C2—C3	−176.8 (7)	I2—C19—C20—C21	−176.0 (9)
I1—C1—C6—C5	176.3 (7)	I2—C19—C24—C23	178.2 (9)
C1—C2—C3—C4	−0.8 (15)	C19—C20—C21—C22	−3 (2)
C2—C1—C6—C5	−4.3 (15)	C20—C19—C24—C23	0.1 (18)
C2—C3—C4—C5	−1.8 (15)	C20—C21—C22—C23	1 (2)
C2—C3—C4—C7	178.6 (10)	C20—C21—C22—C25	177.6 (11)
C3—C4—C5—C6	1.4 (15)	C21—C22—C23—C24	1 (2)
C3—C4—C7—C8	3.7 (16)	C21—C22—C25—C26	−168.3 (16)
C4—C5—C6—C1	1.7 (15)	C22—C23—C24—C19	−2 (2)
C4—C7—C8—C9	−173.6 (9)	C22—C25—C26—C27	−178.2 (12)
C5—C4—C7—C8	−175.9 (9)	C23—C22—C25—C26	8 (3)
C6—C1—C2—C3	3.9 (14)	C24—C19—C20—C21	2.1 (17)
C7—C4—C5—C6	−179.0 (9)	C25—C22—C23—C24	−174.7 (12)
C7—C8—C9—C10	169.9 (10)	C25—C26—C27—C28	168.0 (16)
C7—C8—C9—C14	−8.6 (15)	C25—C26—C27—C32	−11 (2)
C8—C9—C10—C11	−173.1 (10)	C26—C27—C28—C29	−176.4 (10)
C8—C9—C14—C13	173.0 (9)	C26—C27—C32—C31	177.1 (12)
C9—C10—C11—C12	−0.6 (17)	C27—C28—C29—C30	−2.1 (16)
C10—C9—C14—C13	−5.5 (14)	C28—C27—C32—C31	−1.8 (17)
C10—C11—C12—C13	−4.3 (16)	C28—C29—C30—C31	0.7 (14)
C10—C11—C12—C15	171.5 (11)	C28—C29—C30—C33	−176.3 (9)
C11—C12—C13—C14	4.2 (15)	C29—C30—C31—C32	0.0 (15)
C11—C12—C15—C16	−80.3 (15)	C29—C30—C33—C34	−142.2 (10)
C11—C12—C15—C17	43.5 (18)	C29—C30—C33—C35	97.2 (11)
C11—C12—C15—C18	163.3 (11)	C29—C30—C33—C36	−23.5 (14)
C12—C13—C14—C9	0.6 (15)	C30—C31—C32—C27	0.5 (18)
C13—C12—C15—C16	95.2 (15)	C31—C30—C33—C34	41.0 (14)
C13—C12—C15—C17	−140.9 (13)	C31—C30—C33—C35	−79.7 (12)
C13—C12—C15—C18	−21.2 (16)	C31—C30—C33—C36	159.7 (10)
C14—C9—C10—C11	5.5 (14)	C32—C27—C28—C29	2.6 (16)
C15—C12—C13—C14	−171.6 (10)	C33—C30—C31—C32	177.0 (10)

### Hydrogen-bond geometry (Å, º)

Cg2 and Cg4 are the centroids of the C9–C14 and C27–C32 rings, respectively.

**Table:**

*D*—H···*A*	*D*—H	H···*A*	*D*···*A*	*D*—H···*A*
C1—I1···*Cg*2^i^	2.09 (1)	3.63 (1)	5.676 (10)	166 (1)
C19—I2···*Cg*4^ii^	2.10 (1)	3.57 (1)	5.526 (11)	154 (1)

Symmetry codes: (i) −*x*+1, −*y*, *z*−1/2; (ii) −*x*+3/2, *y*−1, *z*+1/2.
