# Supplementary figures and images for: Crystal structure of (E)-1-(4-tert-butyl­phen­yl)-2-(4-iodo­phen­yl)ethene
Source: Acta Crystallogr E Crystallogr Commun. 2015 Apr 15;71(Pt 5):o309–10. doi: 10.1107/S2056989015007185 (PMC4420064; doi:10.1107/S2056989015007185)

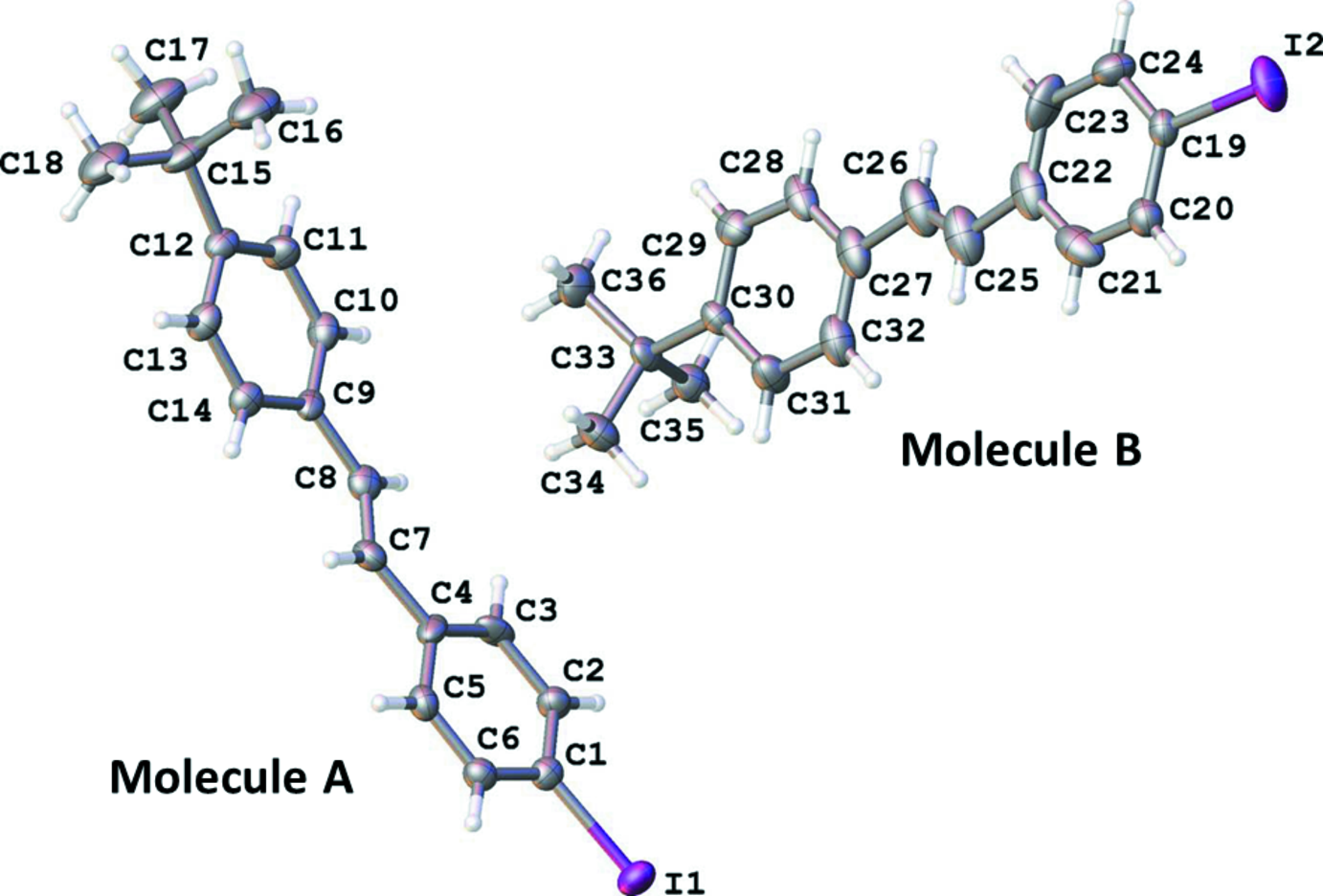

Supplement: Supplementary file 3 [file e-71-0o309-fig1.tif]

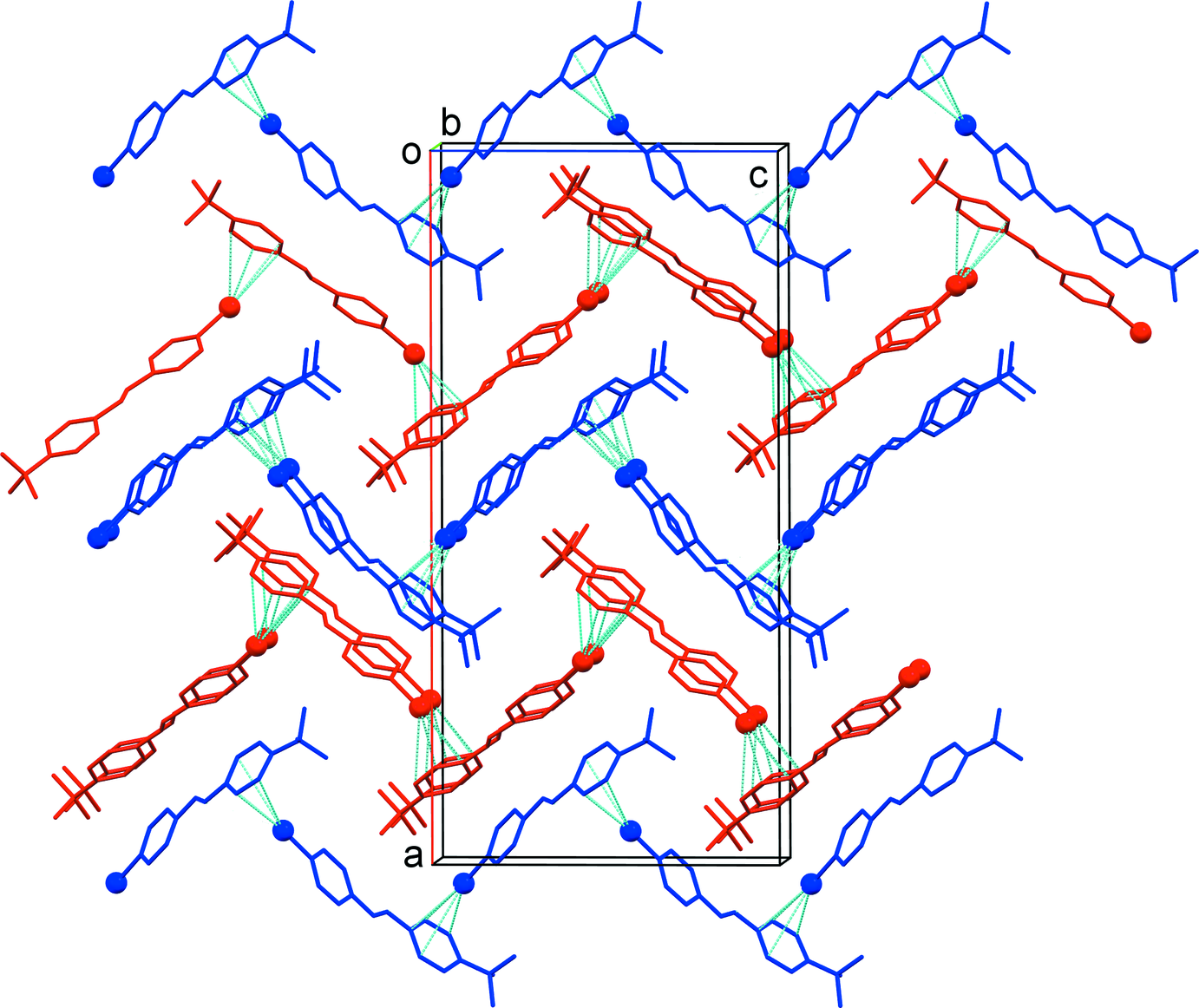

Supplement: Supplementary file 4 [file e-71-0o309-fig2.tif]

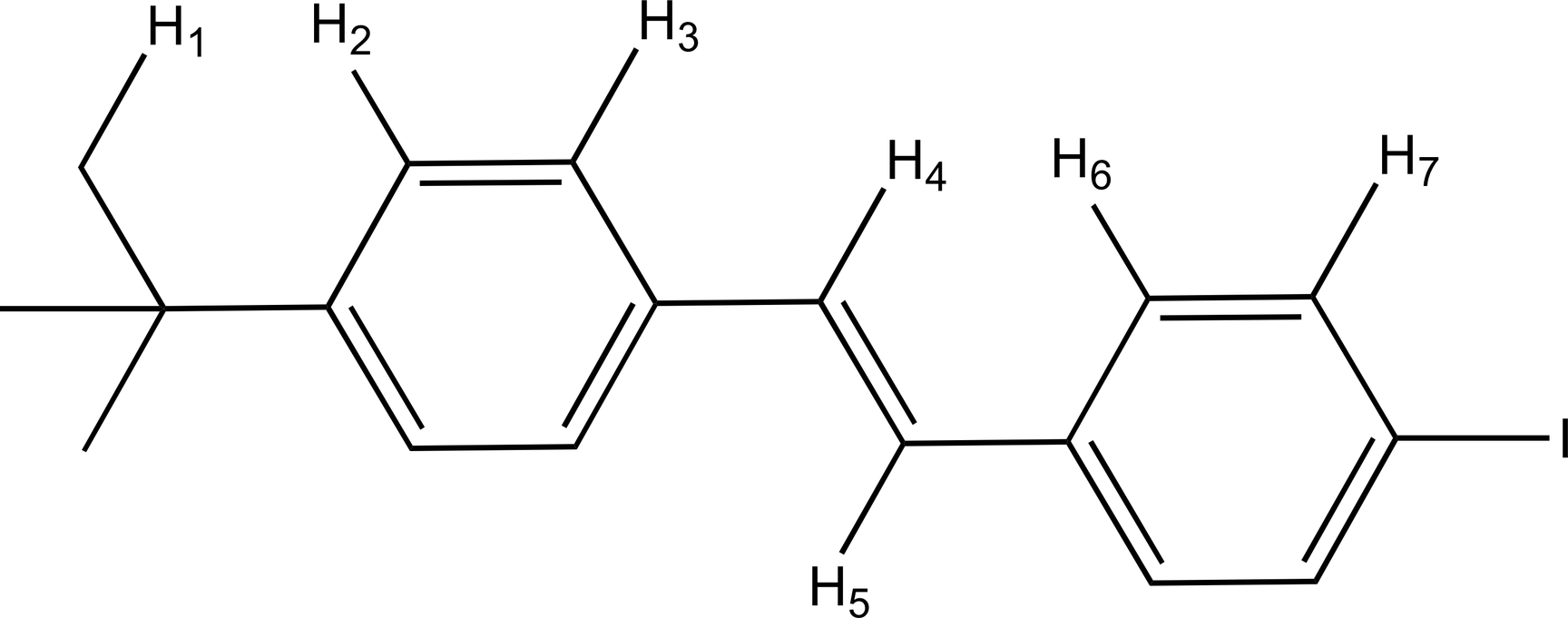

Supplement: Supplementary file 5 [file e-71-0o309-fig3.tif]
